# Evaluation and clinical implications of interactions between compound Danshen dropping pill and warfarin associated with the epoxide hydrolase gene

**DOI:** 10.3389/fphar.2023.1105702

**Published:** 2023-05-04

**Authors:** Xixi Chen, Xurui Zuo, Yingqiang Zhao, Yuhong Huang, Chunxiao Lv

**Affiliations:** ^1^ Department of Clinical Pharmacology, Second Affiliated Hospital of Tianjin University of Traditional Chinese Medicine, Tianjin, China; ^2^ Department of Cardiology, Qingdao Hiser Hospital Affiliated of Qingdao University (Qingdao Traditional Chinese Medicine Hospital), Qingdao, China; ^3^ Department of Cardiology, Second Affiliated Hospital of Tianjin University of Traditional Chinese Medicine, Tianjin, China

**Keywords:** warfarin, compound Danshen dripping pill, epoxide hydrolase gene (EPHX1), microsomal epoxide hydrolase (mEH), pharmacokinetic, pharmacodynamics

## Abstract

**Introduction:** In clinical practice, warfarin is often combined with Compound Danshen dripping pill (CDDP) for the treatment of cardiovascular diseases. However, warfarin has a narrow therapeutic index, wide interindividual variability (genetic and non-genetic factors), and is susceptible to drug-drug interactions. Our previous study indicated that CDDP might interact with warfarin in individuals with the epoxide hydrolase gene (EPHX1; single-nucleotide polymorphism: rs2292566) A/A subtype. We sought to clarify the interaction between CDDP and warfarin associated with EPHX1 in a comprehensive and accurate manner.

**Methods:** Here, EPHX1 A and EPHX1 G cell lines were established. Expression of microsomal epoxide hydrolase (mEH), vitamin K epoxide reductase (VKOR), and vitamin K-dependent clotting factors (FII, FVII, FIX, FX) was measured by western blotting upon incubation with CDDP and warfarin. mEH activity was evaluated by measuring the transformation of epoxyeicosatrienoic acids into dihydroxyeicosatrienoic acids. Then, healthy volunteers (HVs) with the EPHX1 A/A genotype were recruited and administered warfarin and CDDP to investigate the pharmacokinetics and pharmacodynamics of warfarin.

**Results:** CDDP combined with warfarin could decrease expression of mEH and VKOR, and increase protein expression of FII, FVII, FIX, and FX, in EPHX1 A cells. CDDP could slightly influence the pharmacokinetics/pharmacodynamics of warfarin in HVs with the EPHX1 A/A genotype.

**Discussion:** Rational combination of CDDP and warfarin was safe with no risk of bleeding, but the therapeutic management is also needed. The clinical study is posted in the China Clinical Trial Registry (ChiCTR190002434).

## 1 Introduction

Use of herbs and herbal products is widespread and increasing steadily worldwide (Reddy et al., 2021). Sales of herbal supplements in the USA were more than $8 billion in $2017 and $9.6 billion in 2019 (Suroowan et al., 2021). In 2020, the world trade in medicinal plants was $138.35 billion and is expected to reach $218.94 billion by 2026 (Cnudde et al., 2022; Hossain et al., 2022). Furthermore, an estimated one-third of adults in developed countries and approximately 75%–80% of the population in developing countries consume herbal medicines for primary healthcare (Claure-Del Granado and Espinosa-Cuevas, 2021).

However, the increasing use of medicinal plants has attracted growing concern with regard to their efficacy and safety if combined with conventional medicines. Herbal plants contain multiple phytochemicals that act upon different targets and pathways. Hence, there is a risk of interactions if another drug is administered simultaneously or successively (Gomes et al., 2021). Therefore, comprehensive evaluation of interactions between herbal drugs and conventional drugs is essential.

Warfarin has a narrow therapeutic index, wide variability in efficacy between individuals, and can interact with other drugs (Mar et al., 2022). It acts by inhibiting vitamin K epoxide reductase (VKOR) and disruption of the synthesis of biologically active forms of vitamin K-dependent (VKD) clotting factors (FII, FVII, FIX, FX), as well as regulatory factors (protein C, protein S) (Wang et al., 2021; Abdelnabi et al., 2022). Thus, warfarin is employed commonly for the prevention and treatment of the coagulopathic and thromboembolic disorders associated with cardiac valve-replacement, atrial fibrillation, and other cardiovascular conditions (Zhang et al., 2021).

In China, the most prevalent herbal medicine used against cardiovascular diseases is Compound Danshen dripping pill (CDDP). It was first launched by the China Food and Drug Administration in 1995, and has been approved by the Australian Therapeutic Goods Administration. It is undergoing a phase-III clinical trial overseen by the US Food and Drug Administration (Yan et al., 2021; Hu et al., 2022).

In clinical practice, warfarin often combines with CDDP, and many scholars have reported on this interaction (Chu et al., 2011; Zhang et al., 2020; Zhuang et al., 2021; Muyambo et al., 2022). However, most reports have focused on animals, healthy volunteers (HVs), or pharmacometrics models to predict this interaction. Moreover, whether CDDP interacts with warfarin has been reported inconsistently.

Recently, we recruited patients with coronary heart diseases and atrial fibrillation to investigate the interaction between CDDP and warfarin (Lv et al., 2017; Lv et al., 2019). CDDP did not influence the stable dose, stable concentration, or International Normalized Ratio (INR) of warfarin in most patients. However, the INR seemed to be higher in patients with the epoxide hydrolase gene (EPHX1; single nucleotide polymorphism (SNP): rs2292566) A/A subtype than in those with other EPHX1 subtypes. There were 33 SNPs identified in the EPHX1 gene and mainly two variant EPHX1 alleles (A, G) (Saito, et al., 2001). The people carrying the A allele was 30% for Caucasian and nearly 50% for Chinese. Interestingly, previous reports indicated EPHX1 polymorphisms were not related with warfarin dosage in Caucasian (Ciccacci, et al., 2011; Özer, et al., 2013). However, EPHX1 A>G could explain about 43% of the interindividual variability in warfarin dosage requirements for Chinese (Gu et al., 2010). EPHX1 is the encoding gene of microsomal epoxide hydrolase (mEH), a crucial enzyme that catalyzes epoxides reactions (Lin et al., 2020). The potential associations between mEH genotypes and the susceptibility to certain diseases have been investigated in various laboratories (Saito, et al., 2001). Moreover, mEH is an important component of VKOR, and EPHX1 variants may alter mEH activity, thereby influencing the efficacy of warfarin therapy (Cain et al., 1998). On the other hand, CDDP contained multiple phytochemicals, which mostly included phenolic acids, saponins and tanshinones (Liu, et al., 2014). These multiple ingredients would undergo complex metabolic processes in the body, which might effect the mEH activity and furtherly influence the coagulation function of warfarin *in vivo*.

Thus, we wished to clarify the interaction between CDDP and warfarin associated with EPHX1 in a comprehensive and accurate manner. Hence, transfected EPHX1 A cells and transfected EPHX1 G cells were adopted to explore the interaction between CDDP and warfarin, and the result was verified using HVs with the EPHX1 A/A genotype.

## 2 Materials and methods

### 2.1 Evaluation of the interaction between CDDP and warfarin associated with EPHX1 a cells and EPHX1 G cells

#### 2.1.1 Preparation of EPHX1 A cells and EPHX1 G cells

**FIGURE 1:**
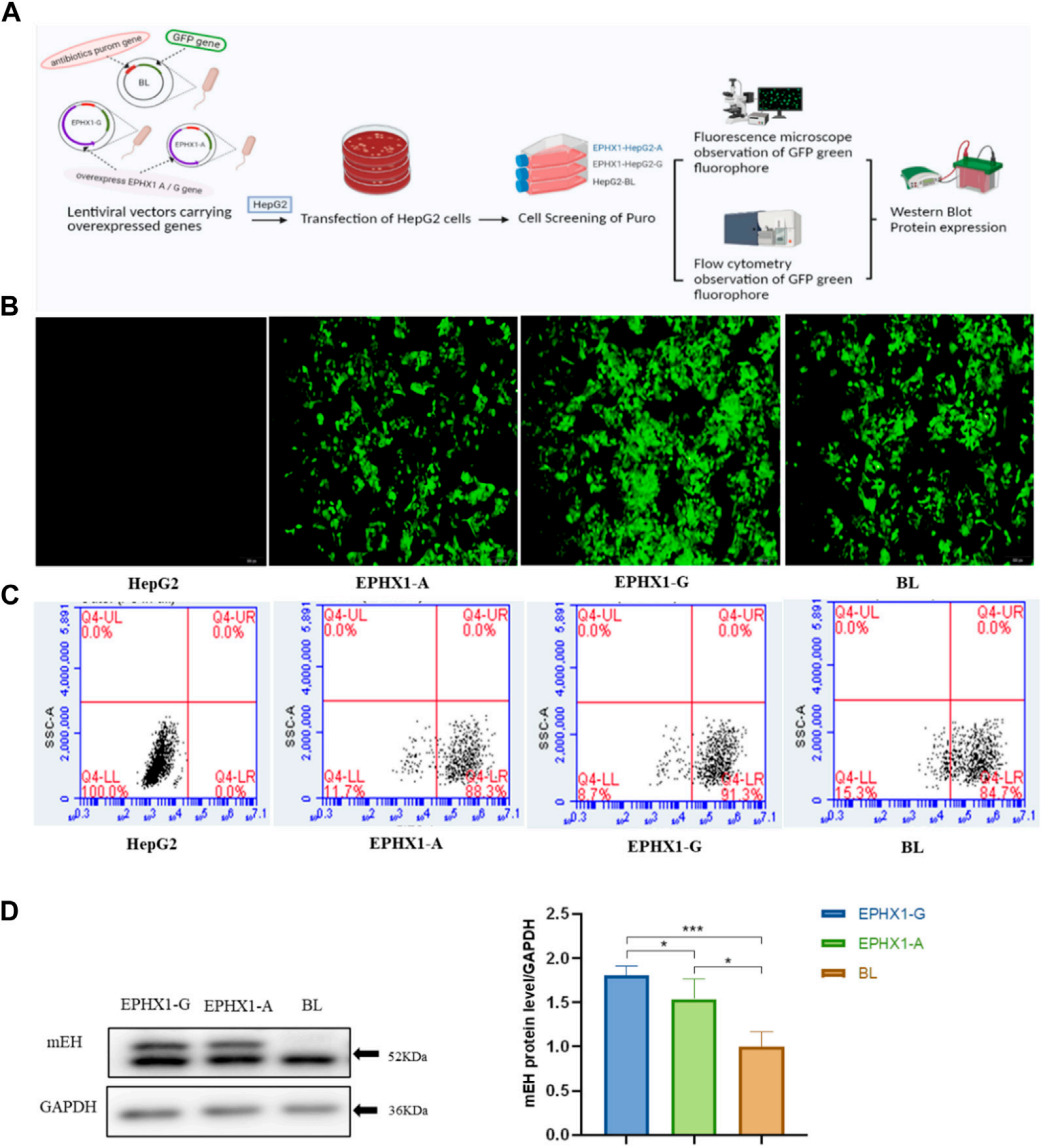
Establishment and verification of transfected EPHX1 A cells and transfected EPHX1 G cells. **(A)** Flowchart for establishment and verification of transfected EPHX1 A cells and transfected EPHX1 G cells. **(B)** We used a fluorescence microscope (×10 objective lens) to evaluate transfected EPHX1 A cells, transfected EPHX1 G cells, and transfected BL cells. Transfected EPHX1 A cells, transfected EPHX1 G cells, and transfected BL cells had abundant light-green spots, and HepG2 cells had no fluorescence. **(C)** Fluorescence ratio for transfected EPHX1 A cells, transfected EPHX1 G cells, and transfected BL cells according to flow cytometry. **(D)** Protein expression of mEH in transfected EPHX1 A cells, transfected EPHX1 G cells, and transfected BL cells detected by western blotting.

Human hepatocarcinoma (HepG2) cells were purchased from the Chinese Academy of Medical Sciences & Peking Union Medical College (Beijing, China). They were cultured in Dulbecco’s modified Eagle’s medium supplemented with 10% fetal bovine serum and 1% penicillin/streptomycin in an atmosphere of 5% CO_2_ at 37°C. EPHX1 A cells or EPHX1 G cells were prepared as shown in Figure 1A. Briefly, HepG2 cells were transduced with overexpressed EPHX1 A lentiviral particles or EPHX1 G lentiviral particles, which were incorporated within an antibiotic puromycin gene and a green fluorescent protein plasmid that could co-express with the target protein, at a multiplicity of infection of 10. For comparison, we set a negative control group (BL) of transfected cells that lacked an overexpressed EPHX1 gene.

Transfected cells were cultured in medium containing puromycin (2 μg/mL) to select stably transfected cells. Subsequently, a microscope (IX71; Olympus, Tokyo, Japan) equipped with a bandpass filter of 488 nm was used to capture images of intracellular green fluorescence. Then, we used a flow cytometer (Accuri C6; Becton Dickinson Biosciences, San Jose, CA, United States) to measure the proportion of intracellular green fluorescence from the green fluorescent protein that integrated with transfected cells. Finally, western blotting was applied to measure mEH expression in transfected cells.

#### 2.1.2 Determination of the incubation time and concentration of CDDP and warfarin in cell lines

CDDP was manufactured by Tianjin Tasly Group (Tianjin, China). Warfarin was obtained from Orion Corporation (Espoo, Finland). Transfected cells were plated evenly on a 96-well cell-culture plate with ∼8,000 cells/well. They were cultured for 24 h at 37°C. Then, they were treated with CDDP or warfarin at 0.0625–8.00 mg/mL for 24 h or 72 h. Subsequently, to each well was added 10 µL of 3-(4,5-Dimethylthiazol-2-yl)-2,5-diphenyltetrazolium bromide solution (5 mg/mL), and cells were allowed to incubate for an additional 4 h in an atmosphere of 5% CO_2_ at 37°C. Then, the cells were centrifugated and the supernatant was retained. Finally, the blue-purple crystals were dissolved using dimethyl sulfoxide (150 µL) with agitation for 10 min. The absorbance of each well was determined at 490 nm. Cell viability (Supplementary Figure S1) was calculated according to the formula:

$$Cell\;viability={absorbance}_{experiment}/{absorbance}_{control}\times 100\%$$

#### 2.1.3 Determination of expression of VKD coagulation factors

We wished to measure expression of mEH, VKOR, FII, FVII, FIX, and FX. Whole-cell extracts were prepared using radio immunoprecipitation assay buffer (Solarbio, Beijing, China) incubated on ice for 30 min. Cell lysates were centrifuged (13,000 rpm, 25 min, 4°C). The protein concentration of the supernatant was determined by a bicinchoninic acid protein assay kit (CWBIO, Beijing, China). Lysates (10 µg) were subjected to sodium dodecyl sulfate-polyacrylamide gel electrophoresis using 10% gels. Proteins were transferred to polyvinylidene fluoride (PVDF) membranes (Thermo Fisher Scientific, Waltham, MA, United States). PVDF membranes were blocked with 10% non-fat dried milk at room temperature (Solarbio) for 1.5 h and incubated overnight at 4°C with antibody directed against mEH (1:1000 dilution; Santa Cruz Biotechnology, Houston, TX, United States), VKOR, FII, and FIX (1:1000; Abcam, Cambridge, United Kingdom), FVII (1:8,000; Abcam), FX (1:10,000; Abcam), and glyceraldehyde 3-phosphate dehydrogenase (GAPDH; 1:10,000; Solarbio) in TBS-T buffer (Tris (20 mM), pH 7.6, NaCl (100 mM), 0.1% Tween-20) for immunoblotting. After incubation for 2 h at room temperature with secondary anti-rabbit immunoglobulin-G antibody conjugated with horseradish peroxidase (1:5000; Solarbio), proteins were visualized by an electrochemiluminescence detection system. Western-blotting signals were quantified using ImageJ 1.6.0.20 (US National Institutes of Health, Bethesda, MD, United States) and were normalized to GAPDH expression.

#### 2.1.4 Enzyme activity of mEH

mEH catalyzes the hydrolysis of lipophilic epoxides to their corresponding dihydrodiols (Oesch et al., 1971; Gautheron and Jeru, 2021). Free epoxyeicosatrienoic acids (EETs) are epoxides that can be degraded further by mEH into dihydroxyeicosatrienoic acids (DHETs) (McReynolds et al., 2020) (Figure 3E). Thus, the metabolic efficiency of the substrate (DHETs/EETs) (Arand et al., 1999) was determined to characterize the catalytic activity of mEH on the endogenous substrates of 14,15-EET. Valpramide was selected as an inhibitor of mEH.

The concentration of 14,15-EET and 14,15-DHET was measured by liquid chromatography tandem-mass spectrometry (LC-MS/MS). The separation and detection of 14,15-EET (319.2→219.2) and 14,15-DHET (337.2→207.0) was undertaken in an Acquity ultra-performance liquid chromatography (UPLC) system (Waters, Milford, MA, United States) with a Triple Quad 5500 MS/MS detector (AB Sciex, Foster City, CA, United States). Detailed information and method validation are shown in Supplementary Materials.

Transfected EPHX1 A cells, transfected EPHX1 G cells, and transfected BL cells were seeded 1 day before treatment with a substrate or inhibitor. Then, the cell-culture medium was replaced with complete medium containing CDDP (0.5 mg/mL) and warfarin (0.125 mg/mL) (CDDP + War group), or warfarin (0.125 mg/mL) (War group), or valpramide (100 mg/mL) (valpramide group, VPD). After incubation with 14,15-EET, 60 µL of the cell culture medium was harvested at 0, 0.25, 0.5, 1, 1.5, 2, and 3 h after addition of 14,15-EET. Subsequently, to the harvested cell-culture medium was added 3 µL of chloramphenicol (internal standard) and extracted with ethyl acetate (180 µL) with vortex-mixing for 3 min and centrifugation (14,000 rpm, 10 min, room temperature). The supernatant was evaporated under a stream of nitrogen and re-dissolved by 50% methanol/water (60.0 µL), then 2.00 µL was injected into the LC-MS/MS system.

### 2.2 Evaluation of the interaction between CDDP and warfarin associated with the EPHX1 A/A genotype in HVs

#### 2.2.1 Design of the clinical trial

This was an open-label, three-period, controlled before-and-after trial (China Clinical Trial Registry: ChiCTR1900024344). It was conducted at the Second Affiliated Hospital within Tianjin University of Traditional Chinese Medicine (Tianjin, China). The protocol was approved by the ethics committees of the Second Affiliated Hospital within Tianjin University of Traditional Chinese Medicine. Written informed consent was provided by all participants. This trial was in accordance with the Declaration of Helsinki 1964 and its later amendments.

Men and women aged 18–45 years participated in this trial if they were healthy and the EPHX1 A/A genotype was confirmed. The key exclusion criteria were coagulation disorders or allergies to drugs.

#### 2.2.2 Treatment in the clinical trial

Our trial comprised three periods. In the first period, warfarin (3 mg, p. o.) was taken once a day. After 3 days, individuals entered the second period, in which CDDP (10 pills, p. o., t.d.s.) was administered for 5 days. Finally, participants were treated with a combination of warfarin (3 mg, p. o.) and CDDP (10 pills, p. o.) in the third period.

During the first and third period, 15 blood samples (2-mL each time) were collected for the concentration analysis of warfarin at 0.5, 1, 1.5, 2, 3, 4, 6, 8, 12, 24, 36, 48, and 72 h after administration. Eight blood samples were collected for the detection of coagulation indices before as well as 12, 24, 30, 36, 48, 54, and 72 h after administration. Moreover, blood samples were taken from participants once a day before CDDP administration for assays of coagulation indices to ensure safety.

#### 2.2.3 Markers of the pharmacokinetics and pharmacodynamics of warfarin

The plasma concentration of S-warfarin and R-warfarin was measured by LC-MS/MS, as described previously (Lv et al., 2017; Lv et al., 2019). The pharmacokinetic parameters of warfarin were calculated using the “non-compartmental analysis” tool of Phoenix WinNonlin 6.4 (Certara, Princeton, NJ, United States). The parameters were the peak plasma concentration (C_max_), time to reach C_max_ (t_max_), the area under the curve from time 0 h–144 h (AUC_0–144_), the area under the curve from time 0 to infinity (AUC_0–∞_), half-life (t_1/2_), volume of distribution (V_d_), and clearance (CL).

In addition, the coagulation indices of prothrombin time (PT), INR, thrombin time (TT), activated prothrombin time (APTT), and fibrinogen level were detected according to the manufacturer instructions of assay kits in the clinical laboratory of the Second Affiliated Hospital within Tianjin University of Traditional Chinese Medicine.

#### 2.2.4 Enzyme-linked immunosorbent assays (ELISAs) to determine the content of VKD coagulation factors in HVs with the EPHX1 A/A genotype

Blood samples of the trough concentration and peak concentration of warfarin were used to measure the content of mEH, VKOR, FII, FVII, FIX, FX, and FIIa using the respective human ELISA kits. mEH and VKOR kits were from Lanso (Shanghai, China). FII and FX kits were from Cusabio (Wuhan, China). FVII and FIX kits were from Ruifan (Shanghai, China). The FIIa kit was from Abcam. FIIa is also called thrombin and not exist *in vitro*.

### 2.3 Statistical analyses

Statistical analyses were carried out using SPSS 22.0 (IBM, Armonk, NY, United States). Graphs were plotted using Prism 8.0 (GraphPad, La Jolla, CA, United States). ANOVA was used to compare the results of transfected EPHX1 A cells or transfected EPHX1 G cells by western blotting. For HVs with the EPHX1 A/A genotype, *p*-values (two-sided) were calculated and paired *t*-tests were used for statistical analyses if the data passed the normality (Shapiro–Wilk) test, or the Wilcoxon matched-pairs test if the distribution was not normal. Results are the mean ± SD for continuous variables with a normal distribution. *p* < 0.05 was considered significant.

## 3 Results

### 3.1 Evaluation of the interaction between CDDP and warfarin associated with EPHX1 A cells and EPHX1 G cells

#### 3.1.1 Establishment and validation of transfected EPHX1 A cells and transfected EPHX1 G cells

Transfected EPHX1 A cells and transfected EPHX1 G cells were established to explore differences between EPHX1 A and EPHX1 G subtypes *in vitro* (Figure 1A). Subsequently, the green fluorescence located in transfected EPHX1 A cells, transfected EPHX1 G cells, and transfected BL cells was observed by fluorescence microscopy, and non-transfected HepG2 cells showed no fluorescence (Figure 1B). The efficiency of transfection was examined by flow cytometry: it was 88.3% in transfected EPHX1 A cells, 91.3% in transfected EPHX1 G cells, and 84.7% in BL cells (Figure 1C). Western blotting demonstrated that mEH expression in transfected EPHX1 A cells and transfected EPHX1 G cells was increased compared with that in transfected BL cells (*p* < 0.05) (Figure 1D). These results suggested that transfected EPHX1 A cells and transfected EPHX1 G cells had been constructed successfully.

#### 3.1.2 CDDP combined with warfarin influenced expression of mEH, VKOR, and VKD clotting factors (FII, FVII, FIX, FX) in transfected EPHX1 A cells

**FIGURE 2:**
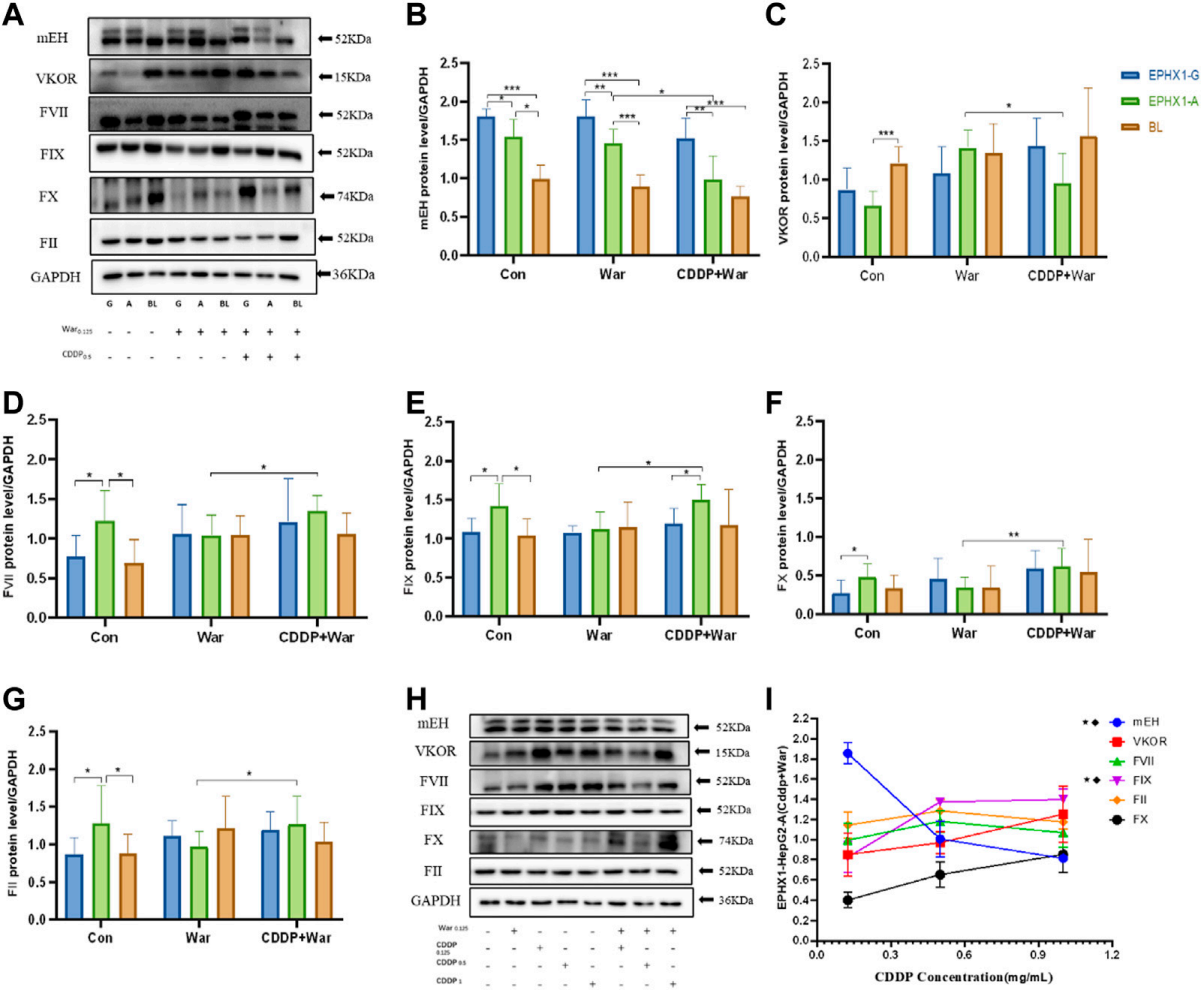
Protein expression of mEH, VKOR, FVII, FIX, FX, and FII. **(A)** Protein expression of mEH, VKOR, FVII, FIX, FX, and FII was measured using western blotting. **(B–G)** Protein expression of mEH, VKOR, FVII, FIX, FX, and FII normalized to that of GAPDH. **(H)** Protein expression of mEH, VKOR, FII, FVII, FIX, and FX at various concentrations of CDDP combined with warfarin. **(I)** Line chart of protein expression of mEH, VKOR, FVII, FIX, FX, and FII normalized to that of GAPDH at various concentrations of CDDP combined with warfarin. ★Significant difference when comparing the CDDP (0.125 mg/mL) group with the CDDP (0.5 mg/mL) group (*p* < 0.05); ◆Significant difference when comparing the CDDP (0. 5 mg/mL) group with the CDDP (1 mg/mL) group (*p* < 0.05). Data are the mean ± SD (*n* ≥ 3). *p < 0.05, **p < 0.01 and ***p < 0.001. EPHX1-A: transfected EPHX1 A cells; EPHX1 **(G)** transfected EPHX1 G cells; BL: transfected BL cells; Con: incubated with cell culture; War: incubated with warfarin; CDDP + War: incubated with CDDP and warfarin.

For the control group, mEH expression (Figures 2A, B) in transfected EPHX1 A cells was reduced significantly, protein expression of FII, FVII, FIX, and FX (Figures 2A, D–G) was increased, and VKOR expression was not significantly different, compared with transfected EPHX1 G cells. These data suggested that expression of mEH, FII, FVII, FIX, and FX in transfected EPHX1 A cells and transfected EPHX1 G cells was significantly different.

Protein expression of mEH and VKOR (Figures 2A–C) was decreased in the CDDP + War group (*p* < 0.05) compared with that in the War group in transfected EPHX1 A cells. Protein expression of FII, FVII, FIX, and FX Figures 2A, D–G) in the CDDP + War group was increased significantly compared with that in the War group (*p* < 0.05) in transfected EPHX1 A cells, and there was no significant difference between the two groups for transfected EPHX1 G cells and transfected BL cells (*p* > 0.05). Thus, various concentrations of CDDP (0.125, 0.5, 1 mg/mL) combined with warfarin (0.125 mg/mL) were incubated in transfected EPHX1 A cells to assess the interaction with CDDP. Expression of mEH and FIX was significantly different (*p* < 0.05), whereas the other indicators did not show a significant difference between the three concentrations of CDDP (Figures 2H, I). Expression of all the indicators did not change continually as the CDDP dose increased.

#### 3.1.3 CDDP combined with warfarin influenced the hydrolytic activity of mEH

**FIGURE 3:**
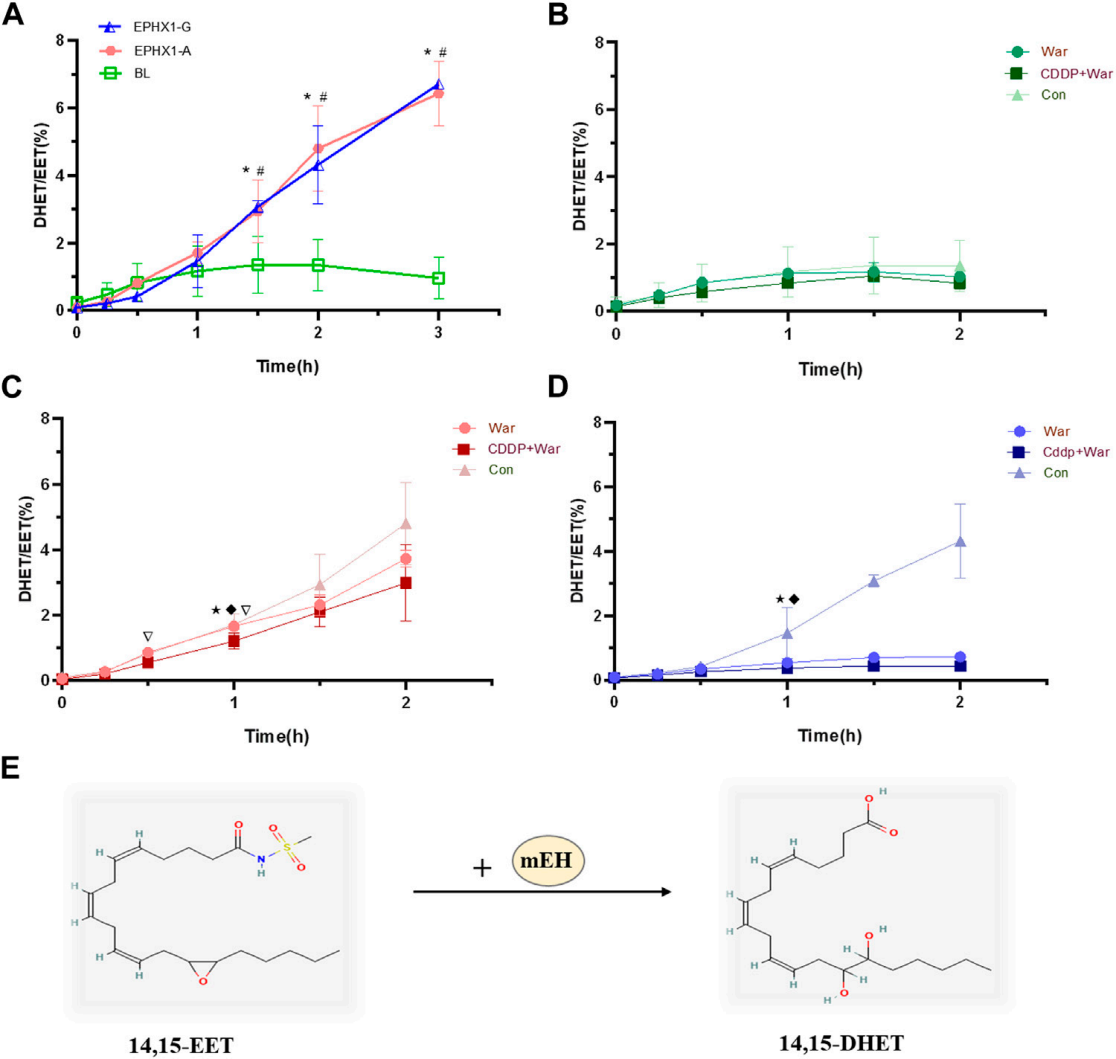
Metabolic efficiency curve of 14,15-EET in transfected EPHX1 A cells, transfected EPHX1 G cells, and transfected BL cells. **(A)** Comparison of the metabolic efficiency of 14,15-EET in transfected EPHX1 A cells, transfected EPHX1 G cells, and transfected BL cells without drug administration. Effect of warfarin, valpramide, and CDDP in combination with warfarin on the metabolic efficiency of 14,15-EET in transfected BL cells **(B)**, transfected EPHX1 A cells **(C)** and transfected EPHX1 G cells **(D)**. **(E)** mEH metabolizes EET to DHET. Data are the mean ± SD (*n* ≥ 3). *Significant difference when comparing transfected EPHX1 A cells to transfected BL cells (*p* < 0.05). #Significant difference when comparing transfected EPHX1 G cells to transfected BL cells (*p* < 0.05). ★Significant difference when comparing the CDDP + War group with the VPD group (*p* < 0.05); ◆Significant difference when comparing the War group with the VPD group (*p* < 0.05). ▽Significant difference when comparing the CDDP + War group with the War group (*p* < 0.05).

CDDP combined with warfarin might affect mEH expression in transfected EPHX1 A cells, but not in transfected EPHX1 G cells or transfected BL cells. We wished to ascertain if a combination of CDDP and warfarin affected the enzyme activity of mEH. Thus, the substrate of mEH (14,15-EET) was chosen and metabolic efficiency was calculated. The curves for the metabolic efficiency of 14,15-EET in transfected EPHX1 A cells and transfected EPHX1 G cells were higher than that for transfected BL cells (*p* < 0.05) (Figure 3A).

The metabolic efficiency of 14,15-EET at all time points in the CDDP + War group was lower than that represented in the War group for transfected EPHX1 A cells or transfected EPHX1 G cells, and the difference was significant at 1 h (*p* < 0.05) (Figures 3B, C, D). When comparing with VPD group at 1 h, CDDP + War group in transfected EPHX1 A cells and G cells were both significantly decreased (*p* < 0.05) (Figures 3C, D). These findings suggested that mEH was the primary enzyme involved in the metabolism of 14,15-EET in transfected EPHX1 A cells and transfected EPHX1 G cells, and that CDDP combined with warfarin might inhibit the enzyme activity of mEH to decrease the hydrolysis of the substrate (14,15-EET), but there was no significant difference between transfected EPHX1 A cells and transfected EPHX1 G cells.

### 3.2 Evaluation of the interaction between CDDP and warfarin associated with the EPHX1 A/A genotype in HVs

#### 3.2.1 Study population

**FIGURE 4:**
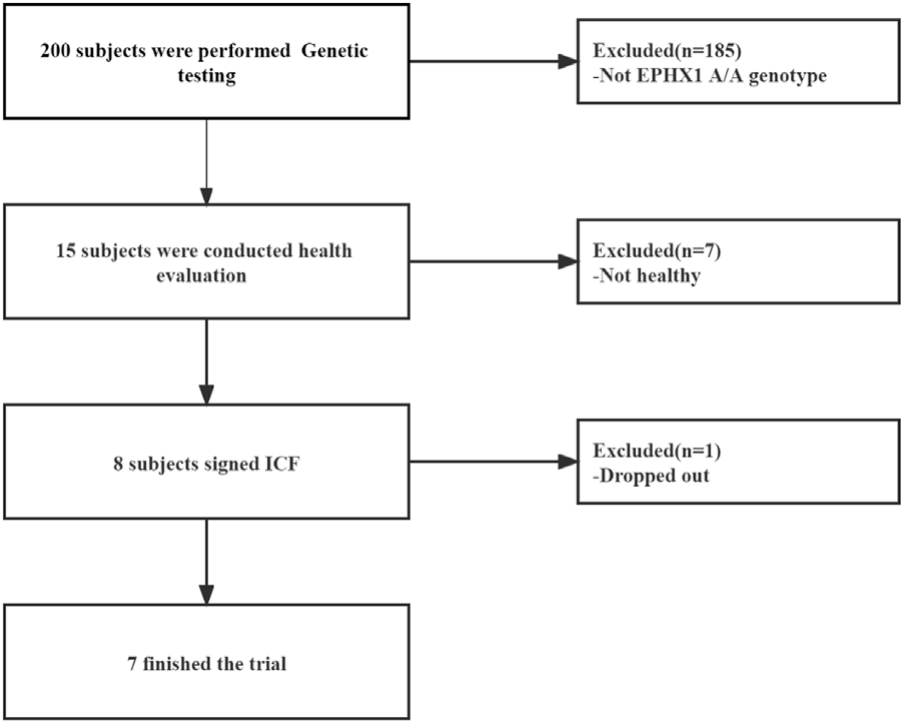
Flowchart of our clinical study.

From March 2019 to September 2019, 15 HVs with the EPHX1 A/A genotype were selected from the Second Affiliated Hospital within Tianjin University of Traditional Chinese Medicine. Eight HVs were enrolled and seven [four males; mean age = 29.25 (range, 22–34) years] completed our clinical trial (Figure 4).

#### 3.2.2 CDDP combined with warfarin influenced the pharmacokinetic profile of warfarin in HVs with the EPHX1 A/A genotype

**FIGURE 5:**
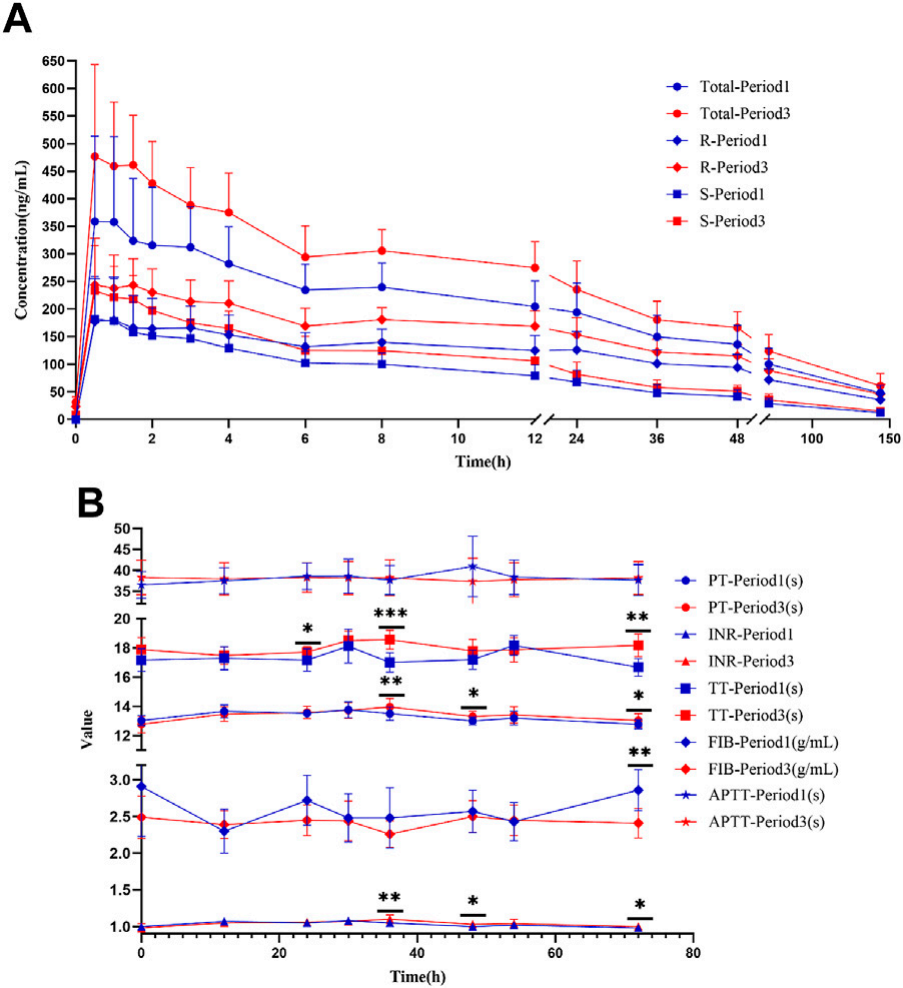
Warfarin concentration and coagulation indices in HVs with the EPHX1 A/A genotype. **(A)** Concentration–time curves of warfarin. Total: concentration of total warfarin; R: R-warfarin concentration; S: S-warfarin concentration. **(B)** Line chart of the four coagulation indices. PT: prothrombin time; INR: International Normalized Ratio of prothrombin time; TT: thrombin time; FIB: fibrinogen; APTT: activated prothrombin time. *p < 0.05, **p < 0.005, ***p < 0.001.

A concentration-time curve was plotted (Figure 5A). The plasma concentration (ng/mL) of warfarin 72 h after administration in the first period and third period was determined, and was 100.6 ± 28.50 and 124.1 ± 29.64 for warfarin, 71.94 ± 19.92 and 88.73 ± 21.10 for R-warfarin, and 28.65 ± 9.578 and 35.32 ± 10.50 for S-warfarin, respectively. The plasma concentration (ng/mL) 144 h after administration in the first period and third period was 47.93 ± 14.07 and 60.97 ± 22.67 for warfarin, 35.61 ± 11.23 and 45.84 ± 17.89 for R-warfarin, and 12.33 ± 3.713 and 15.12 ± 6.294 for S-warfarin, respectively. Hence, the warfarin concentration 72 h and 144 h after administration was higher in the third period than that in the first period (Supplementary Table S3, Figure 5A).

The pharmacokinetic parameters of warfarin were calculated using Phoenix WinNonlin. There was a significant difference (*p* < 0.05) in the AUC, C_max_, CL, and V_d_ between the first period and third period, whereas t_1/2_ and T_max_ did not show a significant difference (Table 1). However, drug elution in HVs was incomplete in the second period. The residual drug concentration was converted into a dose, and pharmacokinetic parameters were re-calculated (Supplementary Table S4): there was no significant difference in V_d_ or CL between the two periods. Thus, CDDP might influence the AUC and C_max_ of warfarin in HVs with the EPHX1 A/A genotype, but would not influence warfarin elimination.

**TABLE 1:** Pharmacokinetic parameters of Warfarin in EPHX1 A/A healthy subjects.

Ingredient	PK parameter	The first period	The third period	*p*-Value
		Mean ± SD (ng/mL)	Mean ± SD (ng/mL)	
Warfarin	t_1/2_ (h)	64.77 ± 7.072	66.42 ± 16.11	0.795
	AUC_0-144_ (ng/h/mL)	17443 ± 4477	21818 ± 4570	0.000***
	AUC_0-∞_ (ng/h/mL)	21984 ± 5883	28081 ± 8220	0.003**
	V_d_ (mL)	13393 ± 3036	10437 ± 1239	0.016*
	CL (mL/h)	144.9 ± 36.87	114.3 ± 30.01	0.004**
	C_max_ (ng/mL)	403.4 ± 148.2	535.8 ± 112.4	0.001***
	T_max_ (h)	1.857 ± 1.435	0.7143 ± 0.3934	0.094
R-Warfarin	t_1/2_ (h)	68.87 ± 9.371	73.76 ± 23.80	0.583
	AUC_0-144_ (ng/h/mL)	11630 ± 2938	14594 ± 3088	0.000***
	AUC_0-∞_ (ng/h/mL)	15255 ± 4280	19962 ± 6805	0.008*
	V_d_ (mL)	20505 ± 4506	16283 ± 2164	0.022*
	CL (mL/h)	210.3 ± 57.13	164.8 ± 51.16	0.003**
	C_max_ (ng/mL)	204.6 ± 71.38	273.9 ± 58.10	0.001***
	T_max_ (h)	1.857 ± 1.435	0.7143 ± 0.3934	0.094
S-Warfarin	t_1/2_ (h)	55.84 ± 5.110	54.57 ± 8.595	0.777
	AUC_0-144_ (ng/h/mL)	5812 ± 1632	7223 ± 1727	0.001***
	AUC_0-∞_ (ng/h/mL)	6808 ± 1890	8476 ± 2455	0.004**
	V_d_ (mL)	37594 ± 9569	28630 ± 3437	0.017*
	CL (mL/h)	466.0 ± 109.7	374.1 ± 82.69	0.009*
	C_max_ (ng/mL)	199.0 ± 76.90	261.9 ± 54.50	0.002**
	T_max_ (h)	1.500 ± 1.414	0.714 ± 0.393	0.199

#### 3.2.3 CDDP combined with warfarin influenced coagulation indices in HVs with the EPHX1 A/A genotype

PT, INR, and TT were clearly increased at 36 and 72 h in the third period when compared with those in the first period, and the differences were significant (Figure 5B). There were significant differences in PT and the INR at 48 h, TT at 48 h, and the fibrinogen level 72 h after administration between the two periods. A significant difference was not observed in APTT at the various time points, or PT, the INR, TT, or fibrinogen level at 0, 12, 30, and 54 h between the two periods.

#### 3.2.4 CDDP combined with warfarin influenced the content of VKDs coagulation factors in HVs with the EPHX1 A/A genotype

The content of mEH, VKOR, FVII, FIX, FX, FII, and FIIa in HVs with the EPHX1 A/A genotype was measured. Samples were chosen from the trough concentration (C_min_) and peak concentration (C_max_) time points of warfarin as measured by ELISAs.

**FIGURE 6:**
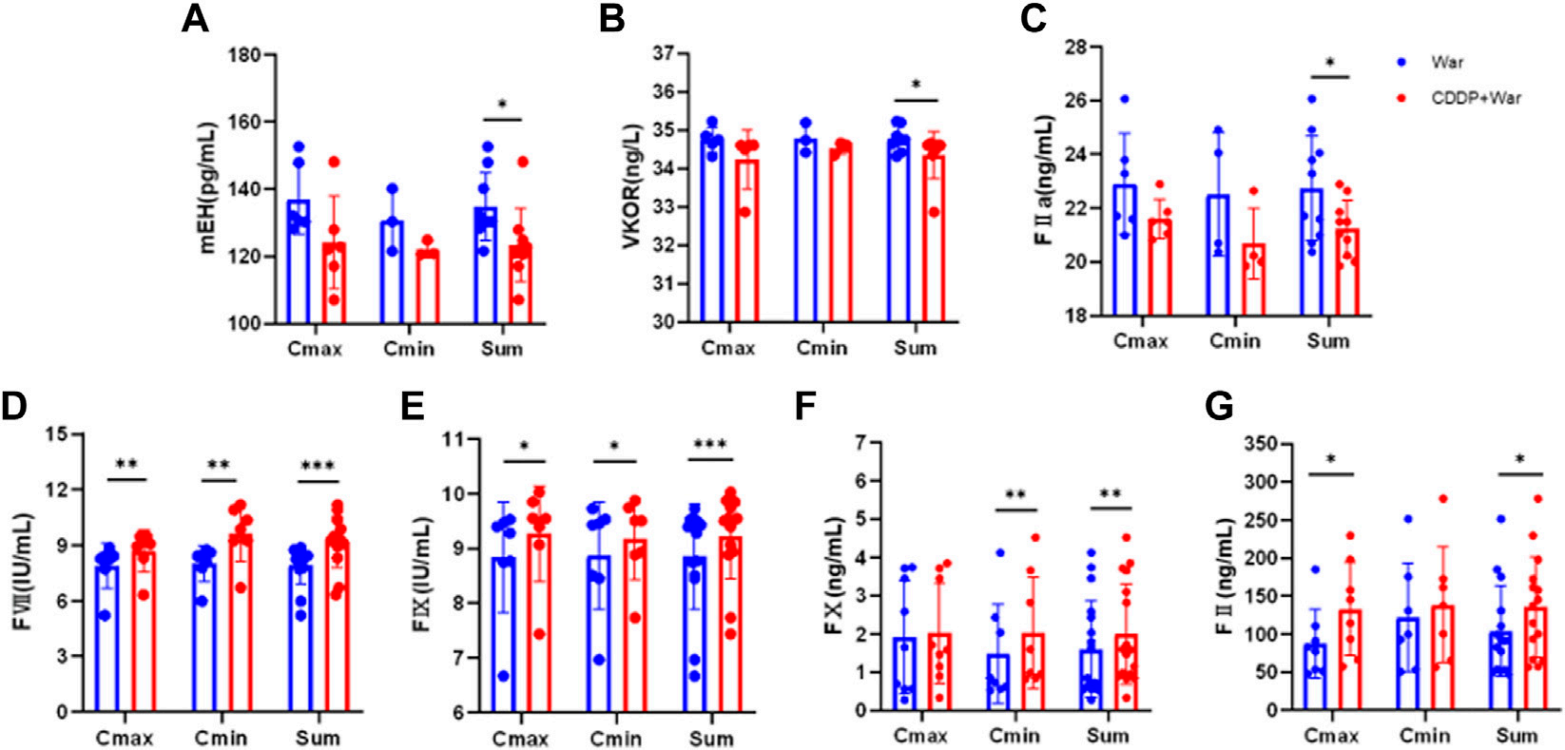
Content of mEH, VKOR, FVII, FIX, FX, FII, and FIIa in HVs with the EPHX1 A/A genotype. **(A–G)** Blue dot: warfarin group (first period); red dot: CDDP + War group (third period). ELISAs were undertaken to measure the content of mEH, VKOR, FVII, FIX, FX, FII, and FIIa in HVs with the EPHX1 A/A genotype. *p < 0.05, **p < 0.01 and ***p < 0.001. Data are the mean ± SD (*n* ≥ 3).

The content of all indices did not show a significant difference between C_min_ and C_max_ in the War group or CDDP + War group (Figure 6). Thus, C_min_ and C_max_ were combined to obtain a “Sum” group. In the latter, the content of mEH, VKOR, and FIIa was decreased significantly, and the content of FVII, FIX, FX, and FII was increased significantly, in the third period compared with those in the first period (*p* < 0.05). These results suggested that CDDP combined with warfarin might affect the content of mEH, VKOR, FVII, FIX, FX, FII, and FIIa in HVs with the EPHX1 A/A genotype compared with those taking only warfarin.

## 4 Discussion

In recent years, formulations based on traditional Chinese medicine (TCM) have been used widely in clinical therapy and are, in general, considered safe (Shi et al., 2022). However, an increased risk of interactions between TCM formulations and western medicines has been documented.

Warfarin and CDDP are often used in combination for treatment of cardiovascular diseases (Chu et al., 2011). Previously, we focused on the EPHX1 A/A subtype (Lv et al., 2017; Lv et al., 2019). In the present study, overexpressing EPHX1 cells were established and applied to evaluate the interaction between CCDP and warfarin. Then, *in vitro* results were verified in HVs with the EPHX1 A/A genotype.

mEH was first purified from rabbit liver (Watabe and Kanehira, 1970) and then characterized in human liver (Oesch, 1974). mEH plays a prominent part in human hepatic physiology because of its enzyme activity (Gautheron and Jeru, 2021). In hepatocytes, the primary function of mEH is detoxifying many xenobiotic compounds (El-Sherbeni and El-Kadi, 2014). It catalyzes the hydrolysis of various endogenous fatty-acid epoxides to their respective diols (Edin and Zeldin, 2021), including the metabolism of endocannabinoid EETs to DHETs. Moreover, mEH is an essential component in VKOR, which suggests that modulation of mEH activity might contribute to blood coagulation and further influence VKD coagulation factors (Guenthner et al., 1998). On the other hand, the multiple ingredients from CDDP would finally eliminate from the body. The mEH acted as a detoxification role and could convert compounds with epoxides into a more water-soluble metabolites which would eliminate easier. During these metabolic pathways, some ingredients or metabolites from CDDP would influence the expression or activity of mEH, and finally leading to a content fluctuation of coagulation factors.

In humans, the EPHX1 (SNP: rs2292566) gene includes A/A, A/G, and A/A subtypes. A genetic polymorphism is a potential determinant of mEH activity, and contributes to differences among individuals (Nguyen et al., 2013). The different genes of EPHX1 (A/G) led to a significant difference in expression of mEH, FII, FVII, FIX, and FX (Figure 2), which might influence biological functions. Moreover, the warfarin dose is assumed to be associated with SNP rs2292566 in the EPHX1 gene, so the warfarin dose must be managed carefully before the initiation of warfarin therapy (Pautas et al., 2010).

CDDP combined with warfarin in EPHX1 A cells could reduce expression of mEH and VKOR, inhibit the enzyme activity of mEH (Figure 3), increase protein expression of FII, FVII, FIX, and FX (Figure 2), reduce activation of FII (FIIa), and lead to lower activity of coagulation factors in HVs with the EPHX1 A/A genotype (Figure 6). However, a significant difference was not observed in EPHX1 G cells. Therefore, CDDP worked in tandem with warfarin in HVs with the EPHX1 A/A genotype to restrain the catalytic activity of mEH and VKOR (Hassett et al., 1994; Shen et al., 2017) that is required to sustain blood coagulation. Thus, we speculated that a combination of CDDP and warfarin might prolong PT and elevate the INR in HVs with the EPHX1 A/A genotype.

Warfarin combined with CDDP impacted the blood concentrations and pharmacokinetic parameters of warfarin in HVs with the EPHX1 A/A genotype, which were increased by ∼30%. The trough concentration of warfarin in the third period (Supplementary Table S3) suggested that drug elution was incomplete from the first period. Then, residual drugs were converted to a dose of drug administered, and pharmacokinetic parameters were recalculated. Ultimately, there was no significant difference in V_d_ and CL between the two periods after this adjustment (Supplementary Table S4).

The INR is used routinely to monitor the level of anticoagulation elicited by warfarin (Al Ammari et al., 2020). According to certain clinical trials as well as criteria set by the Division of Microbiology and Infectious Diseases within the American National Institute of Allergy and Infectious Diseases, a 1.01–1.25-fold increase in the upper limit of normal in coagulation indices is considered to denote a “mild” difference (Kovacevic et al., 2019; Akdag et al., 2020). In our study, the increase in PT, the INR, and fibrinogen level was <10% after a combination of CDDP and warfarin, and all INR values were within 0.91–1.18 (a normal value for a healthy person is 0.8–1.2). Thus, CDDP combined with warfarin had a very low impact on the INR and did not show a bleeding risk in HVs with the EPHX1 A/A genotype. Conversely, expression of mEH, VKOR, FII, FVII, FIX, and FX plateaued gradually with increasing concentrations of CDDP (Figure 2I), indicating that VKD clotting factors (FII, FVII, FIX, FX) would not be inhibited infinitely with increasing concentrations of CDDP. Thus, the dose safety of CDDP was shown to be in a combination of CDDP and warfarin. Collectively, these observations suggest that the interactions might occur if warfarin is co-administered with CDDP in HVs with the EPHX1 A/A genotype, but the effect on the pharmacokinetics/pharmacodynamics of warfarin is slight with no risk of bleeding.

There are some limitations in this study. The dose and ratio of warfarin and CDDP in cell experiment were not inconsistent with the clinical dose. For the cell experiment, the concentration of warfarin and CDDP were determined by the MTT assay (Supplementary Figure S1) and the survival rate of cells was the major concern. However, the clinical efficacy and safety were the essential issue for the patients in clinical. In this study, the results of cell experiment were further verified by the clinical trial, which indicated that the cell experiment with different dose from the clinical could also provide some guidance for the clinical research. On the other hand, the medication time of the subjects administrated of warfarin and CPPD was relatively short, and the subjects were all healthy person. Thus, although there was no risk of bleeding for the healthy person, the therapeutic management is also needed if warfarin is combined with CDDP in patients, especially those exposed to risks of over-anticoagulation and bleeding.

## 5 Conclusion

CDDP combined with warfarin might reduce expression of mEH and VKOR, and increase protein expression of FII, FVII, FIX, and FX, in EPHX1 A cells. In HVs with the EPHX1 A/A genotype, CDDP could influence the pharmacokinetics/pharmacodynamics of warfarin slightly, but the combination is safe with no risk of bleeding, but this is still a warning signal for patients with EPHX1 A/A.

## Data Availability

The original contributions presented in the study are included in the article/Supplementary Material, further inquiries can be directed to the corresponding authors.
