# Supplementary material for: Evaluation and clinical implications of interactions between compound Danshen dropping pill and warfarin associated with the epoxide hydrolase gene
Source: Front Pharmacol. 2023 May 4;14:1105702. doi: 10.3389/fphar.2023.1105702 (PMC10192880; doi:10.3389/fphar.2023.1105702)

**Supplemental Materials**

**1. Determination the incubation time and concentration for CDDP and warfarin in transfected cells by the MTT assay**

The cell growth curve was used to observe CDDP or warfarin effect on transfected EPHX1 A/G cells. As shown in Figure S1, transfected EPHX1 A/G and BL cells’ viability significantly decreased after treatment with CDDP or warfarin for 24 h or 72 h, which were in a dose-dependent manner. With the increasing incubation time and concentration, the cells’ viability was brought down and showed no obvious difference. Thus, the incubation time selected at 24 h. In order to ensure the cell survival radio ≥ 85%, the concentration of warfarin was selected at 0.125 mg/mL, and CDDP was selected at low, medium, and high concentrations (0.125 mg/mL, 0.5 mg/mL and 1 mg/mL) for subsequent experiments.

**2.** **Determination the concentration of 14,15-EET and14,15-DHET by LC-MS/MS**

**2.1 Instrumentation and Chromatographic Conditions**

The ACQUITY ultra-performance liquid chromatography (UPLC) system was equipped with a degasser, an autosampler, and a binary pump (Waters Corporation, Milford, Massachusetts). The Triple Quad 5500 MS/MS system was from AB Sciex (Foster City, California). All the operations, acquisition, and analysis of data were performed by Analyst version 1.6.2 (AB Sciex). An ACQUITY UPLC BEH C18 column (2.1 × 50 mm, 1.7 μm) with mobile phase consisted of phase A (0.01% formic acid (FA) in water) and B (0.01% FA in acetonitrile) (50:50, v/v; isocratic elution). The flow rate was 0.5 mL/min, and the sample injection volume was 2.00 μL. The ion spray voltage was set at -4500 V. The source temperature was set at 600℃, curtain gas, gas 1, and gas 2 (all gases, nitrogen) were set at 30, 55, and 60 psi, respectively. Table S1 listed the quantitative parameters.

**2.2 Preparation of standard solutions**

Preparing stock solutions of 14,15-EET, 14,15-DHET and IS was in ethyl alcohol at the concentration of 100.0 μg/mL. The standards were further diluted by adding the blank cell incubation medium with the corresponding mixed standard solutions to make final concentrations 4.000, 8.000, 40.00, 100.0, 200.0, 400.0, 1000 and 2000 ng/mL for 14,15-EET and 14,15-DHET. Quality control (QC) samples were prepared in the same fashion (4.000, 40.00, and 400.0 ng/mL).

**2.3 Method Validation**

According to the Bioanalytical Method Validation Guidance in the Chinese Pharmacopoeia and the US Food and Drug Administration, selectivity, calibration curve, lower limit of quantitation (LLOQ), accuracy and precision, recovery and matrix effect were determined.

The specificity was comparing areas of analytes in blank plasma from 6 different cell media samples versus the areas of analytes in LLOQ, which determined if there was significant interference at the retention time of 14,15-EET, 14,15-DHET and IS.

The linearity for 14,15-EET and 14,15-DHET with a total of 8 calibration standards (4.000, 8.000, 40.00, 100.0, 200.0, 400.0, 1000, and 2000 ng/mL) were evaluated that was plotted by a calibration model with analytes-to-IS peak area ratios versus the calibration standard concentration. The calibrations were applied from least squares linear regression with a weighted (1/x^2^) factor. Two independent calibration curve runs were included from each run for 3 different analytical batches: *y* = *ax* + *b* was applied for the calibration models, where the concentration of 14,15-EET or 14,15-DHET in medium was *x*, the peak area ratio of analyte/IS was *y* , slope was *a*, and intercept was *b*. LLOQ was determined as the lower limit of quantitation and must satisfy a signal-to-noise ratio >10. The calibration models would be accepted, when the calibration correlation coefficient *r* was >0.99, and the residuals were within ±20% at the LLOQ and 15% at all other calibration levels.

Accuracy was defined as relative error (RE) and precision was defined as relative SD (RSD). The intraday/interday accuracy (which was within ±15%) and precision (which was between 85% and 115%) were conducted at 3 QC levels (low/medium/high, in 6 replicates) within the same run and on 3 consecutive validation runs.

The absolute extraction recoveries of 14,15-EET and 14,15-DHET were also determined at 3 QC levels (low/medium/ high, in 6 replicates), which was calculated by comparing the mean area of 3 QC level samples with the area in freshly prepared unextracted standards.

The matrix effect was evaluated using matrix factor (MF) of 6 lots of matrix. The peak area of 14,15-EET, 14,15-DHET or IS spiked into the treated blank plasma versus the peak area in pure standard solutions was calculated for MFs. The ratio of 14,15-EET or 14,15-DHET MF to IS MF was defined as IS-normalized MF, with acceptable IS-normalized MF (RSD) < 15%.

**2.4 Results**

**2.4.1 Specificity**

The retention time for 14,15-EET, 14,15-DHET or IS were 3.63, 1.48, and 0.38 min, respectively (Figure S2). The areas of analytes in blank plasma from 6 different cell media samples with the areas of analytes in LLOQ were all < 20%. There was no significant interference observed.

**2.4.2 Linearity and LLOQ**

A linear relationship was found when plotting peak area ratios (analyte/IS) against 14,15-EET or 14,15-DHET concentrations. The relationship was found to be linear for 14,15-EET and 14,15-DHET under the concentration range. The mean values for the regression parameters and the correlation coefficient of 14,15-EET were *y* = 0.004 *x* + 0.0064 (*r* = 0.9977); for 14,15-DHET, they were y = 0.0042 x - 0.01 (*r* = 0.9992). The maximum intra-RE of LLOQ in cell media in 3 runs of 14,15-EET and 14,15-DHET was 5.50% and 0.35%, respectively. The inter-RE of LLOQ of 14,15-EET and 14,15-DHET was 4.03% and 0.38% with inter-RSD was 5.50% and 0.35% (Table S2).

**2.4.3 Precision and Accuracy**

The Intra-day and inter-day precision and accuracy of 14,15-EET and 14,15-DHET (Table S2) were well within acceptance criteria (±15%), indicating satisfactory results for accuracy and precision of 14,15-EET and 14,15-DHET. The recoveries (Table S2) of 14,15-EET were 95.80%, 94.31%, 98.48% and 89.55%, 94.31%, 98.48% for 14,15-DHET at three levels. These results indicated that recoveries of 14,15-EET and 14,15-DHET were consistent and not concentration dependent. The matrix effect was calculated at the 3 QC levels by IS-normalized MF and the RSDs in Table S2 were all < 15%.

**Table S1 Selected parameters for multiple-reaction monitoring, declustering potential (DP), entrance potential (EP), collision energy (CE), and cell exit potential (CXP) for 14,15-EET, 14,15-DHET, and the internal standard (IS).**

| Compound | Q1 | Q3 | DP (V) | EP (V) | CE (V) | CXP (V) |
| --- | --- | --- | --- | --- | --- | --- |
| 14.15-EET | 319.2 | 219.2 | -140 | -10 | -15 | -16 |
| 14.15-DHET | 337.2 | 207.0 | -100 | -10 | -25 | -14 |
| Chloramphenicol (IS) | 321.1 | 152.1 | -100 | -10 | -23 | -17 |

**Table S2. Linearity, precision, accuracy, absolute extraction recovery, and matrix effect of 14,15-EET and 14,15-DHET.**

| Variable | 14,15-EET LQC | 14,15-EET MQC | 14,15-EET HQC | 14,15-DHET LQC | 14,15-DHET MQC | 14,15-DHET HQC |
| --- | --- | --- | --- | --- | --- | --- |
| Calibration (*y* = a*x* + b) | *y*=0.004*x*+0.0064 (*r* = 0.9977) | | | *y*=0.0042*x*-0.01(*r* = 0.9992) | | |
| Intraday accuracy RE% | 2.11 | 0.73 | 0.67 | 3.77 | 1.81 | 1.42 |
|  | 5.98 | 1.40 | 1.80 | 5.98 | 1.40 | 1.80 |
|  | 3.70 | 0.87 | 1.08 | 3.70 | 0.87 | 1.08 |
| Intraday precision RSD% | 7.39 | 6.67 | 8.57 | 8.31 | 4.85 | 6.62 |
|  | 9.72 | 8.45 | 9.92 | 5.80 | 8.45 | 12.19 |
|  | 7.10 | 12.93 | 12.19 | 9.72 | 12.93 | 9.92 |
| Interday accuracy RE% | 4.29 | 7.46 | 6.59 | 3.77 | 1.81 | 1.43 |
| Interday precision RSD% | 7.73 | 6.57 | 11.16 | 8.31 | 14.85 | 8.98 |
| Absolute recovery (mean, %) | 95.80 | 94.31 | 98.48 | 89.55 | 94.31 | 98.48 |
| Matrix effect RSD% | 4.74 | 5.01 | 2.93 | 6.80 | 2.55 | 1.38 |

HQC= high quality control; LQC = low quality control; MQC = medium quality control; RE =relative error; RSD= relative SD

**Table S3. Plasma concentration of warfarin in HVs with the EPHX1 A/A genotype.**

| Ingredient | Time (h) | The first period | The third period | *P* value |
| --- | --- | --- | --- | --- |
|  |  | Mean±SD  (ng/mL) | Mean±SD  (ng/mL) |  |
| Warfarin | 0 | 0.0 ± 0.0 | 31.4 ± 9.3 | 0.000*** |
|  | 0.5 | 358.9 ± 154.7 | 476.6 ± 166.9 | 0.017* |
|  | 1 | 358.0 ± 154.5 | 459.4 ± 116.0 | 0.035* |
|  | 1.5 | 323.9 ± 112.9 | 461.1 ± 90.2 | 0.003** |
|  | 2 | 315.9 ± 104.6 | 427.9 ± 76.1 | 0.002** |
|  | 3 | 312.2 ± 73.1 | 388.6 ± 68.1 | 0.006* |
|  | 4 | 282.2 ± 66.8 | 375.3 ± 71.4 | 0.002** |
|  | 6 | 234.6 ± 46.2 | 294.2 ± 56.7 | 0.000*** |
|  | 8 | 239.7 ± 43.6 | 305.7 ± 38.4 | 0.001*** |
|  | 12 | 204.4 ± 46.4 | 274.9 ± 47.2 | 0.000*** |
|  | 24 | 194.0 ± 53.4 | 235.3 ± 51.6 | 0.010* |
|  | 36 | 149.7 ± 38.7 | 180.4 ± 33.7 | 0.002** |
|  | 48 | 136.0 ± 34.5 | 166.4 ± 28.8 | 0.005** |
|  | 72 | 100.6 ± 28.5 | 124.1 ± 29.6 | 0.001*** |
|  | 144 | 47.9 ± 14.1 | 61.0 ± 22.7 | 0.028* |
| R-Warfarin | 0 | 0.0 ± 0.0 | 23.4 ± 7.6 | 0.000*** |
|  | 0.5 | 177.5 ± 77.5 | 243.5 ± 85.0 | 0.012* |
|  | 1 | 179.6 ± 78.1 | 238.0 ± 59.9 | 0.020* |
|  | 1.5 | 166.0 ± 58.1 | 243.4 ± 47.7 | 0.001*** |
|  | 2 | 164.4 ± 54.7 | 230.3 ± 42.3 | 0.001*** |
|  | 3 | 166.0 ± 39.2 | 213.6 ± 39.1 | 0.003** |
|  | 4 | 152.8 ± 36.0 | 210.5 ± 40.4 | 0.001*** |
|  | 6 | 132.1 ± 25.2 | 169.0 ± 32.6 | 0.000*** |
|  | 8 | 140.0 ± 24.0 | 180.9 ± 21.5 | 0.001*** |
|  | 12 | 125.1 ± 27.1 | 168.6 ± 28.0 | 0.000*** |
|  | 24 | 126.3 ± 32.6 | 153.3 ± 31.3 | 0.010* |
|  | 36 | 101.5 ± 24.8 | 122.4 ± 22.2 | 0.001*** |
|  | 48 | 94.4 ± 23.3 | 115.3 ± 20.5 | 0.007* |
|  | 72 | 71.9 ± 19.9 | 88.7 ± 21.1 | 0.001*** |
|  | 144 | 35.6 ± 11.2 | 45.8 ± 17.9 | 0.021* |
| S-Warfarin | 0 | 0.0 ± 0.0 | 8.1 ± 2.4 | 0.000*** |
|  | 0.5 | 181.4 ± 77.2 | 233.1 ± 82.0 | 0.025* |
|  | 1 | 178.4 ± 76.4 | 221.4 ± 56.3 | 0.064 |
|  | 1.5 | 157.9 ± 54.8 | 217.7 ± 43.2 | 0.006* |
|  | 2 | 151.5 ± 50.0 | 197.5 ± 34.4 | 0.004** |
|  | 3 | 146.2 ± 34.1 | 175.0 ± 29.6 | 0.015* |
|  | 4 | 129.4 ± 31.0 | 164.7 ± 31.6 | 0.006* |
|  | 6 | 102.5 ± 21.2 | 125.2 ± 25.0 | 0.001*** |
|  | 8 | 100.1 ± 20.0 | 124.8 ± 18.1 | 0.003** |
|  | 12 | 79.4 ± 19.7 | 106.3 ± 20.7 | 0.000*** |
|  | 24 | 67.7 ± 21.5 | 82.0 ± 21.8 | 0.014* |
|  | 36 | 48.2 ± 14.7 | 58.0 ± 13.8 | 0.007* |
|  | 48 | 41.6 ± 12.2 | 51.1 ± 11.0 | 0.004** |
|  | 72 | 28.7 ± 9.6 | 35.3 ± 10.5 | 0.001*** |
|  | 144 | 12.3 ± 3.7 | 15.1 ± 6.3 | 0.098 |

**Table S4. Pharmacokinetic parameters of warfarin in HVs with the EPHX1 A/A genotype by dose adjustment.**

| Ingredient | PK  parameter | The first period | The third period | *P* value |
| --- | --- | --- | --- | --- |
|  |  | Mean±SD  (ng/mL) | Mean±SD  (ng/mL) |  |
| Warfarin | t_1/2_  (h) | 64.77 ± 7.072 | 66.42 ± 16.11 | 0.795 |
|  | AUC_0-144_  (ng/h/mL) | 17443±4477 | 21818±4570 | 0.000*** |
|  | AUC_0-∞_  (ng/h/mL) | 21984 ± 5883 | 28081 ± 8220 | 0.003** |
|  | V_d_  (mL) | 13393 ± 3036 | 11845 ± 1377 | 0.124 |
|  | CL  (mL/h) | 144.9 ± 36.87 | 129.6 ± 33.38 | 0.056 |
|  | C_max_  (ng/mL) | 403.4 ± 148.2 | 535.8 ± 112.4 | 0.001*** |
|  | T_max_  (h) | 1.857 ± 1.435 | 0.7143 ± 0.3934 | 0.094 |
| R-Warfarin | t_1/2_  (h) | 68.87 ± 9.371 | 73.76 ± 23.80 | 0.583 |
|  | AUC_0-144_  (ng/h/mL) | 11630 ± 2938 | 14594 ± 3088 | 0.000*** |
|  | AUC_0-∞_  (ng/h/mL) | 15255 ± 4280 | 19962 ± 6805 | 0.008* |
|  | V_d_  (mL) | 20505 ± 4506 | 18445 ± 2408 | 0.181 |
|  | CL  (mL/h) | 210.3 ± 57.13 | 186.2 ± 56.70 | 0.044* |
|  | C_max_  (ng/mL) | 204.6 ± 71.38 | 273.9 ± 58.10 | 0.001*** |
|  | T_max_  (h) | 1.857 ± 1.435 | 0.7143 ± 0.3934 | 0.094 |
| S-Warfarin | t_1/2_  (h) | 55.84 ± 5.110 | 54.57 ± 8.595 | 0.777 |
|  | AUC_0-144_  (ng/h/mL) | 5812 ± 1632 | 7223 ± 1727 | 0.001*** |
|  | AUC_0-∞_  (ng/h/mL) | 6808 ± 1890 | 8476 ± 2455 | 0.004** |
|  | V_d_  (mL) | 37594 ± 9569 | 32429 ± 3754 | 0.094 |
|  | CL  (mL/h) | 466.0 ± 109.7 | 423.5 ± 91.18 | 0.124 |
|  | C_max_  (ng/mL) | 199.0 ± 76.90 | 261.9 ± 54.50 | 0.002** |
|  | T_max_  (h) | 1.500 ± 1.414 | 0.714 ± 0.393 | 0.199 |

**Figure S1**


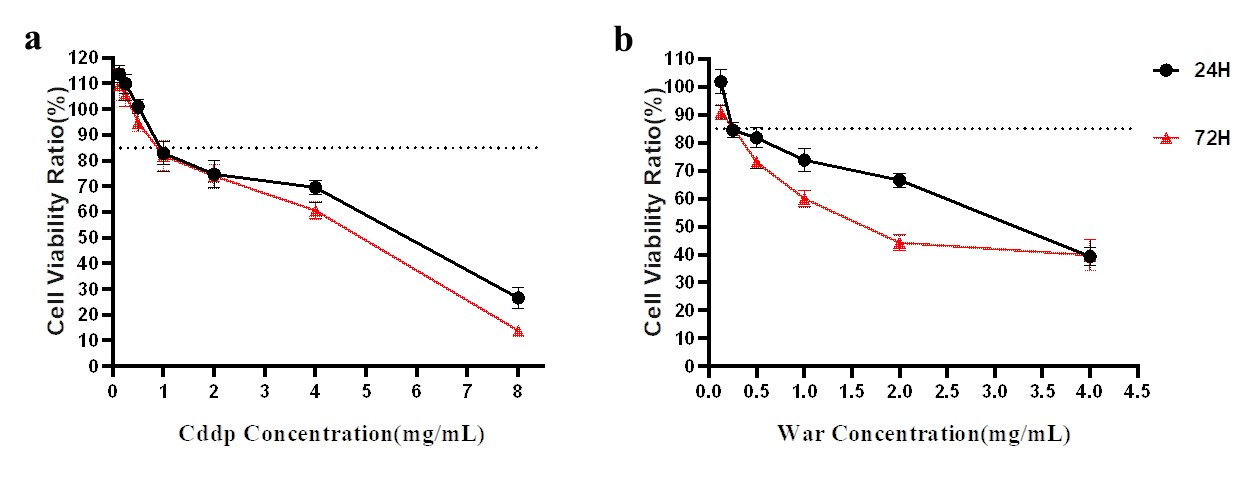


**Figure S2**


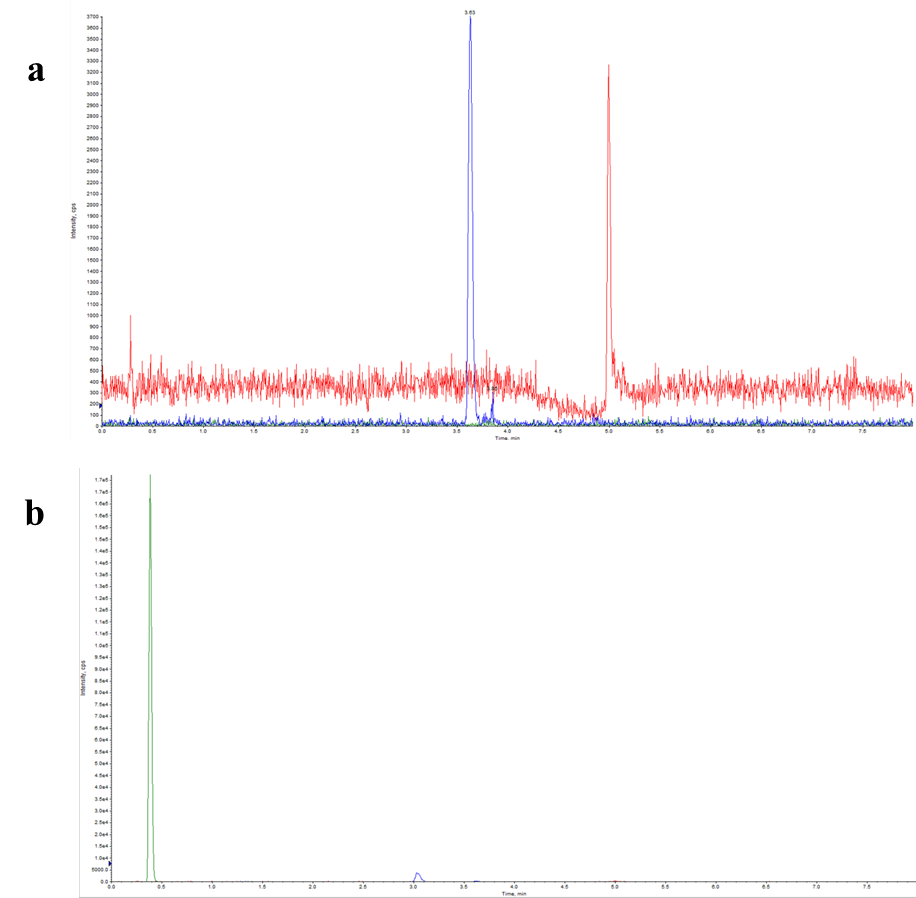

Supplement: Supplementary file 1 [file Table1.DOCX]
